# Pan-Cancer Analysis of KANK2: Clinical and Molecular Insights into Tumor Progression and Therapeutic Implications

**DOI:** 10.7150/jca.105098

**Published:** 2025-01-06

**Authors:** Kai Zhao, Jie Lin, Yongzhi Li, Shenghao Xu, Fei Wang, Yongsheng Yang

**Affiliations:** 1 Department of Hepatobiliary and Pancreatic Surgery, Changchun, Jilin Province, China.; 2 Department of Orthopedics, The Second Hospital of Jilin University, Changchun, Jilin Province, China.; 3 Department of Endocrinology, Weifang People's Hospital, Weifang, Shandong Province, China.

**Keywords:** KANK2, Pan-Cancer Analysis, Tumor Immunity, Bioinformatics, Experimental Verification

## Abstract

Background: KANK2, a gene crucial for cell migration and movement, is implicated in neoplastic and non-neoplastic diseases. This study aimed to analyze KANK2's expression and its diagnostic and prognostic significance across 33 cancers using multiple online databases.

Methods: This study aimed to comprehensively analyze KANK2 in 33 cancers using The Cancer Genome Atlas (TCGA) and Genotype-Tissue Expression (GTEx) database. Multiple web platforms and software were used for data analysis, including R, Cytoscape, HPA, TISIDB, UALCAN, GEO, cBioPortal, STRING, GSCALite, and CancerSEA. WB and qPCR experiments were used to verify the results.

Results: KANK2 is widely expressed in various tissues and has significant diagnostic value in multiple cancers, with AUC values exceeding 0.75 in 13 cancer types. Survival analysis indicated that KANK2 expression is significantly associated with overall survival (OS) and disease-specific survival (DSS) in several cancers. KANK2 expression varied significantly across different molecular and immune subtypes and was associated with specific genetic mutations and DNA methylation patterns. Functional state analysis highlighted correlations with processes such as EMT, angiogenesis, and apoptosis. GSEA identified pathways related to proliferation, migration, and extracellular matrix remodeling. The key interacting proteins were identified by PPI network analysis, and the sensitive drug molecules were found by GSCA database. The results were also confirmed by two GEO datasets and WB and qPCR results.

Conclusion: KANK2 serves as a valuable biomarker for diagnosis and prognosis in various cancers, and its expression is intricately linked to multiple molecular and cellular processes, offering potential therapeutic targets for future research.

## Introduction

Cancer remains a significant global health challenge, highlighting the urgent need for enhanced understanding of its molecular mechanisms^1^. Within this framework, the KANK family, recognized for its roles in cytoskeletal dynamics and cellular signaling, has emerged as a potential contributor to cancer progression^2^-^4^ with KANK2 being particularly influential^5^, ^6^. However, a thorough evaluation of KANK2's expression and function across various cancer types is still missing. The inconsistency in KANK2 expression levels across different tumors emphasizes the necessity for a systematic analysis to elucidate its role in oncogenesis and its viability as a diagnostic and prognostic marker. Furthermore, the relationship between KANK2 and various cancer subtypes—both molecular and immunological—has not been extensively explored.

**Figure 1:**
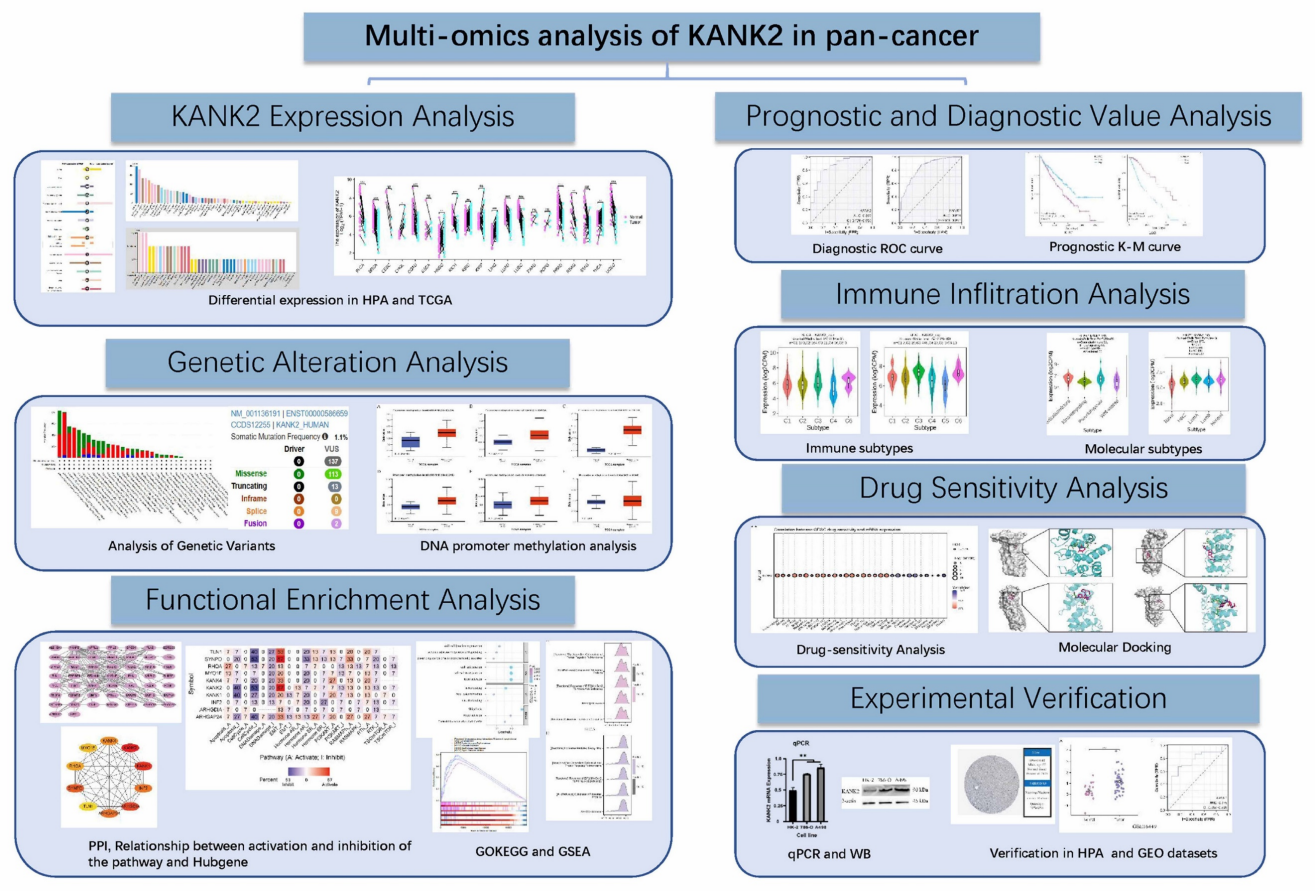
Workflow of the study.

To bridge these gaps, we conducted a comprehensive analysis of KANK2 expression across 33 cancer types, utilizing data from The Cancer Genome Atlas (TCGA) and the GTEx project. We employed rigorous statistical methods to ensure the robustness of our findings, including normalization and transformation of RNA sequencing data. ROC curve analysis was performed to assess KANK2's diagnostic potential, while Kaplan-Meier and Cox regression analyses evaluated its prognostic significance in survival outcomes. Additionally, we investigated KANK2's associations with cancer subtypes using the TISIDB database and explored genetic alterations via cBioPortal. We correlated DNA methylation levels with KANK2 expression and conducted Gene Set Enrichment Analysis (GSEA) to identify enriched pathways. Validation through GEO datasets and protein-protein interaction (PPI) network construction further contextualized KANK2's role in cancer biology. We also examined the therapeutic potential of KANK2 by linking its expression to drug compounds and conducting molecular docking studies, thereby laying a foundation for targeted therapy development. Cell experiments in KIRC cells were performed to verify KANK2 expression (Figure 1, workflow of our study).

In summary, this study provides a comprehensive analysis of KANK2, elucidating its expression patterns across cancers and their clinical implications, thereby positioning KANK2 as a promising candidate for biomarker development and therapeutic intervention in oncology.

## Materials and Methods

### KANK2 expression and datasets acquisition

We utilized the Human Protein Atlas (HPA, https://www.proteinatlas.org/) to summarize the RNA and protein expression profiles of KANK2 in humans. By incorporating data from both The Cancer Genome Atlas (TCGA) and the Genotype-Tissue Expression (GTEx) projects, we obtained KANK2 mRNA expression information from tumor tissues, adjacent non-cancerous tissues, and normal samples spanning 33 cancer types. Any samples lacking gene expression values were excluded, while paired samples were included in further analyses. RNA sequencing data were normalized using Transcripts Per Kilobase Million (TPM) to adjust for sequencing depth and gene length, followed by log2 transformation to stabilize variance for statistical analysis. Specifically, data groups with fewer than three samples or those with zero variance (e.g., SARC, MESO, UVM) were excluded from statistical analysis but were visualized for completeness. All statistical procedures were conducted in R software (version 4.0.2), and the "ggplot2" package (version 3.5.1) was used to generate bar graphs visualizing KANK2 expression across the 33 cancers. The median gene expression level was chosen as the threshold for categorization, and the Wilcoxon rank-sum test was applied to evaluate differences between groups. The analyzed cancer types included: Adrenocortical carcinoma (ACC), Bladder Urothelial Carcinoma (BLCA), Breast invasive carcinoma (BRCA), Cervical squamous cell carcinoma and endocervical adenocarcinoma (CESC), Cholangiocarcinoma (CHOL), Colon adenocarcinoma (COAD), Diffuse Large B-cell Lymphoma (DLBC), Esophageal carcinoma (ESCA), Glioblastoma multiforme (GBM), Head and Neck squamous cell carcinoma (HNSC), Kidney Chromophobe (KICH), Kidney renal clear cell carcinoma (KIRC), Kidney renal papillary cell carcinoma (KIRP), Acute Myeloid Leukemia (LAML), Brain Lower Grade Glioma (LGG), Liver hepatocellular carcinoma (LIHC), Lung adenocarcinoma (LUAD), Lung squamous cell carcinoma (LUSC), Mesothelioma (MESO), Ovarian serous cystadenocarcinoma (OV), Pancreatic adenocarcinoma (PAAD), Pheochromocytoma and Paraganglioma (PCPG), Prostate adenocarcinoma (PRAD), Rectal adenocarcinoma (READ), Sarcoma (SARC), Skin Cutaneous Melanoma (SKCM), Stomach adenocarcinoma (STAD), Testicular Germ Cell Tumors (TGCT), Thyroid carcinoma (THCA), Thymoma (THYM), Uterine Corpus Endometrial Carcinoma (UCEC), Uterine Carcinosarcoma (UCS), and Uveal Melanoma (UVM).

### ROC curve evaluation of KANK2 across 33 cancer types

ROC curves were generated to evaluate the diagnostic potential of KANK2 across the 33 cancer types. The data for constructing these curves was derived from KANK2 mRNA expression levels in cancerous tissues and corresponding normal samples within the TCGA and GTEx databases. The "pROC" package (version 1.18.5) in R was utilized for ROC curve calculations, and plots were generated using the "ggplot2" package. We computed the Area Under the Curve (AUC), along with sensitivity, specificity, positive predictive value, and negative predictive value to assess diagnostic accuracy.

### Survival analysis of KANK2 in 33 cancers

The "survival" package was implemented to perform Kaplan-Meier (K-M) survival analysis, comparing overall survival (OS), disease-specific survival (DSS), and progression-free survival (PFS) between groups with high and low KANK2 gene expression across the 33 cancers. The Cox regression model was employed to determine p-values, while forest plots were constructed using the "survminer" and "ggplot2" packages to visualize hazard ratios (HR), 95% confidence intervals, and p-values.

### KANK2 expression patterns in distinct molecular and immune cancer subtypes

We utilized the "subtype" module of the TISIDB database (http://cis.hku.hk/TISIDB/) to examine the relationship between KANK2 expression and various molecular or immune subtypes in cancers. TISIDB integrates diverse data types to assess interactions between cancer and the immune system^7^, with KANK2 mRNA expression analyzed across immune subtypes such as C1 (wound healing), C2 (IFN-γ dominant), C3 (inflammatory), C4 (lymphocyte depleted), C5 (immunologically quiet), and C6 (TGF-β dominant), as well as different molecular subtypes, including Basal, Her2, lumA, lumB, and HM-SNV. Additionally, we performed immune cell infiltration analysis using the TIMER2.0 database to further explore the association between KANK2 expression and immune cell types. This analysis provided insights into immune infiltration patterns, including correlations with immune checkpoints such as PD-1 and PD-L1, thereby expanding our understanding of KANK2's role in modulating immune responses across different cancers.

### Genetic alteration analysis of KANK2

The cBioPortal (https://www.cbioportal.org/) was queried for data on genetic alterations of KANK2, covering all TCGA Pan-Cancer Atlas Studies^8^-^10^. We examined somatic mutation frequency and genomic information regarding KANK2 mutations in cancers using the "cancer types summary and mutations" and "mRNA vs. study" modules, identifying mutation sites through the "mutations" module.

### Correlation of KANK2 expression with DNA methylation

We conducted an in-depth analysis of TCGA gene expression data using the UALCAN portal (http://ualcan.path.uab.edu/)^11^, focusing on promoter methylation levels of KANK2 across the 33 types of human cancers.

### Correlation analysis of tumor status

CancerSEA (http://biocc.hrbmu.edu.cn/CancerSEA/) facilitated pan-cancer analysis, particularly in tumor state prediction by examining the expression levels of specific genes across various single-cell datasets^12^. We explored the correlation between KANK2 and functional states in 18 cancers, setting a threshold for correlation strength and significance.

### Gene Set Enrichment Analysis (GSEA)

We utilized the "clusterProfiler" package to conduct GSEA, identifying differences in biological pathways between groups with high and low KANK2 expression. Pathways showing significant changes were determined based on a false discovery rate (FDR) < 0.25 and an adjusted p-value < 0.05, with gene set permutations performed 1,000 times. The top five enrichment results were visualized as mountain plots using the "ggplot2" package in R.

### Verification in GEO datasets

Following the diagnostic and prognostic analyses of 33 tumors concerning KANK2, kidney renal clear cell carcinoma (KIRC) exhibited the strongest correlation. Two validation datasets from the GEO database were identified for verification purposes.

### Protein-Protein Interaction (PPI) network analysis of KANK2

The STRING database (https://string-db.org/) was utilized to identify potential protein interactions^13^ with KANK2. The resulting genes underwent PPI network analysis with a confidence score threshold set at > 0.4. Visualization and further analysis were performed using Cytoscape (version 3.8.2), with the cytoHubba plugin identifying key modules and hub genes based on the MCC ranking. Additionally, the Pathlinker plugin was employed to reconstruct signaling pathways from the top 10 hub genes, and expression heat maps alongside Gene Ontology and KEGG analyses were generated concurrently.

### Drugs related to KANK2 and molecular docking

The Gene Set Cancer Analysis (GSCA, https://guolab.wchscu.cn/GSCA/) tool facilitated the identification of gene sets associated with tumor development, progression, or treatment response, along with potential drug targets^14^. GSCAlite queries in the CTRP and GDSC databases predicted drug compounds related to KANK2. The top eight KANK2-related drugs underwent molecular docking and visualization using Chem3D, PyMOL, and other software.

### Experimental verification

KANK2 expression in KIRC, HCC and PAAD was evaluated using quantitative polymerase chain reaction (qPCR), Western blotting (WB) assays and Immunohistochemical staining. Paraffin-embedded sections were processed through dehydration, clearing, and embedding, followed by antigen retrieval, antibody incubation, DAB staining, and microscopy for imaging. Normal and tumor cell lines of KIRC, HCC and PAAD were all generously provided by the Basic Laboratory of Weifang People's Hospital. Cells were cultured in RPMI 1640 medium supplemented with 10% fetal bovine serum (FBS) (Gibco, Grand Island, NY, USA) and 1% penicillin-streptomycin at 37°C in a 5% CO2 incubator. qPCR was used to detect the expression of KANK2 mRNA in the cells. Complementary DNA (cDNA) synthesis followed the manufacturer's instructions for the reverse transcription kit, and quantitative PCR was performed using a fluorescence-based PCR instrument with β-actin as an endogenous control. The following oligonucleotide primers were used: KANK2, forward 5'-GGAGCGCGCAAGGTGT-3' and reverse 5'-AAGTCACTTGAGGGACTGCG-3'; β-actin, forward 5'-TCTGTGTGACACCAACGACC-3' and reverse 5'-TCCTCACATGGGGGAGGTAG-3'. The reaction conditions comprised 45 cycles, starting with denaturation at 95°C for 15 seconds, followed by denaturation at 95°C for 5 seconds and annealing/extension at 60°C for 30 seconds. Relative gene expression levels were calculated using the 2^-ΔΔCt method. For WB analysis, total protein was extracted from normal and tumor cells post-lysis. Protein concentration was determined using a BCA protein assay kit (Beyotime, P0010). Equal protein amounts (50 μg per lane) were separated by 10% SDS-PAGE and subsequently transferred to a polyvinylidene fluoride (PVDF) membrane. The membrane was blocked with 5% milk and incubated with primary antibodies against KANK2 (catalog: a15420; 1:1,000; Abclonal) or anti-β-actin (catalog: AC026; 1:400; Abclonal). After washing, the membrane was treated with a secondary antibody (catalog: SA00001-2; 1:5,000; Proteintech) for 1 hour at room temperature, followed by further washes and visualization using an ECL detection reagent.

## Results

### Pan-cancer expression of KANK2

**Figure 2:**
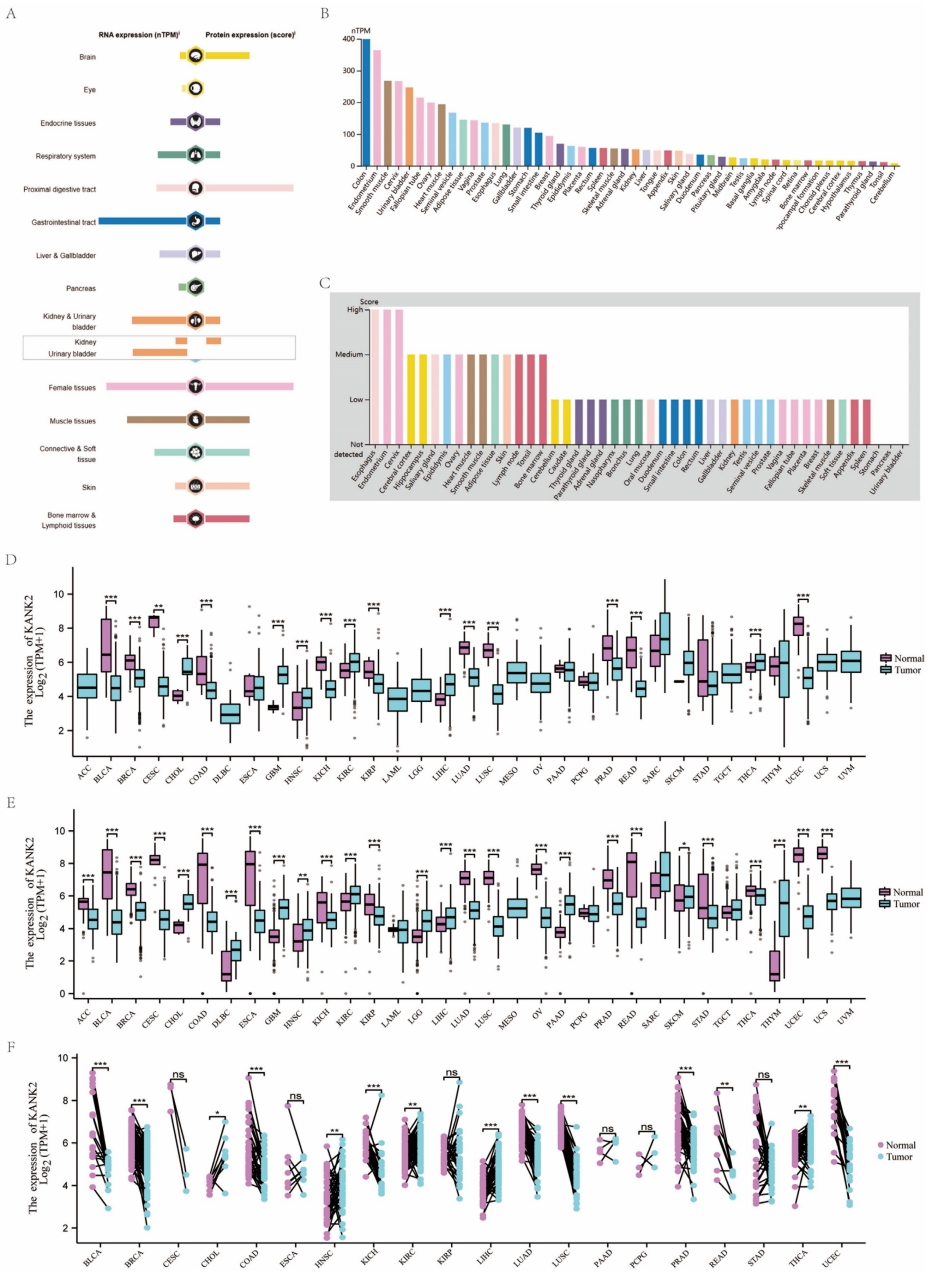
RNA and protein expression profile of KANK2 in human organs and tissues. (A) The summary of KANK2 mRNA and protein expression in human organs and tissues; (B, C) KANK2 mRNA and protein expression summary in different human organs and tissues based on consensus dataset; (D) Expression of KANK2 in 33 cancer tissues and normal tissues in TCGA database. (E) Expression of KANK2 in 33 cases of cancer tissues and adjacent tissues in TCGA+GTEx database; (F) Paired sample analysis of KANK2 mRNA expression between 20 cancers and para-cancerous tissues. ∗p < 0:05, ∗∗p < 0:01, ∗∗∗p < 0:001. ns, Not Significant.

KANK2 is expressed at both mRNA and protein levels across various organs and tissues, as illustrated in Figure 2A. Analysis of a consensus dataset, comprising 436 normal tissues from the Human Protein Atlas (HPA) and 7,568 samples from the Genotype-Tissue Expression (GTEx) project, reveals predominant KANK2 mRNA expression in the colon, endometrium, smooth muscle, cervix, urinary bladder, fallopian tube, ovary, cardiac muscle, seminal vesicle, and adipose tissue (Figure 2B). Protein expression data sourced from the HPA database, which includes information from 144 individuals representing 44 distinct normal tissue types, indicates that KANK2 is primarily expressed in the esophagus, endometrium, cervix, cerebral cortex, hippocampus, salivary gland, epididymis, ovary, cardiac muscle, and smooth muscle (Figure 2C).

The expression levels of KANK2 mRNA across 33 tumor and normal tissue types in the TCGA database are depicted in Figure 2 D-F. Notably, data groups with fewer than three samples or a standard deviation (SD) of zero (specifically SARC, MESO, UVM) were excluded from statistical analysis but were still visualized. In the unpaired sample analysis shown in Figure 2D, a total of 11,123 samples were analyzed, revealing significantly lower KANK2 mRNA expression in bladder cancer (BLCA), breast cancer (BRCA), colon adenocarcinoma (COAD), kidney chromophobe (KICH), kidney renal papillary cell carcinoma (KIRP), lung adenocarcinoma (LUAD), lung squamous cell carcinoma (LUSC), prostate adenocarcinoma (PRAD), rectum adenocarcinoma (READ), and uterine corpus endometrial carcinoma (UCEC) (p < 0.001), as well as in cervical squamous cell carcinoma (CESC) (p < 0.01). Conversely, elevated KANK2 mRNA expression was noted in cholangiocarcinoma (CHOL), glioblastoma multiforme (GBM), head and neck squamous cell carcinoma (HNSC), kidney renal clear cell carcinoma (KIRC), liver hepatocellular carcinoma (LIHC), and pancreatic adenocarcinoma (PAAD) (p < 0.001), with significance in HNSC at p < 0.01. No statistical significance was found in esophageal carcinoma (ESCA), PAAD, pheochromocytoma (PCPG), stomach adenocarcinoma (STAD), and testicular germ cell tumors (TGCT).

When compared to normal tissues, which comprise 18,102 samples, KANK2 mRNA expression was significantly reduced in BLCA, BRCA, CESC, ESCA, COAD, KICH, KIRP, LUAD, LUSC, PRAD, READ, STAD, thyroid carcinoma (THCA), and UCEC (p < 0.001). Elevated expression was observed in CHOL, GBM, KIRC, LIHC, and PAAD (p < 0.001), with significance in HNSC (p < 0.01). In the paired analysis of 11,123 samples (Figure 2E), no significant differences were found in CESC, ESCA, KIRP, PAAD, PCPG, and STAD (p > 0.05). Notably, KANK2 mRNA expression was significantly increased in LIHC (p < 0.001) and HNSC, KIRC, and THCA (p < 0.01), while a decrease was seen in BLCA, BRCA, COAD, KICH, LUAD, LUSC, PRAD, and UCEC (p < 0.001) (Figure 2F).

### The diagnostic value of KANK2

**Figure 3:**
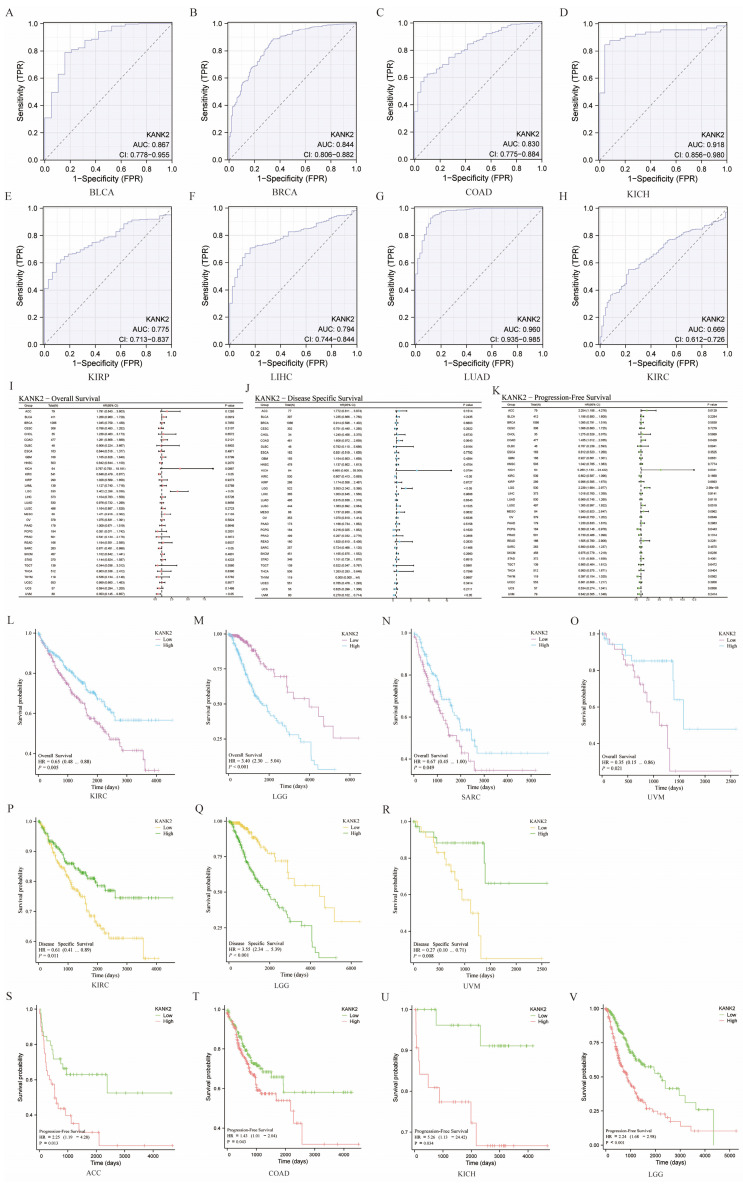
Diagnostic ROC curves and prognostic KM curve for KANK2 in 33 tumors. A-H show the diagnostic ROC curves for 8 of these tumors. (I, J, K) show the forest plots of OS, DSS and PFS of KANK2 in 33 tumors, respectively. (L-O) shows the prognostic curves of KANK2 expression level in KIRC, LGG, SARC, and UVM with OS as the observation index. (P-R) shows the prognostic curve of high and low KANK2 expression with DSS as the observation target in KIRC, LGG, and UVM. (S-V) shows the prognostic curve of high and low KANK2 expression with PFS as the observation target in ACC, COAD, KICH and LGG.

KANK2 demonstrates strong diagnostic potential across various cancers, with an area under the curve (AUC) greater than 0.75 in 13 cancer types, including BLCA (AUC = 0.867), BRCA (AUC = 0.844), CESC (AUC = 0.993), CHOL (AUC = 0.997), COAD (AUC = 0.830), KICH (AUC = 0.918), KIRP (AUC = 0.775), LIHC (AUC = 0.794), LUAD (AUC = 0.960), PRAD (AUC = 0.819), READ (AUC = 0.885), UCEC (AUC = 0.907), and SKCM (AUC = 0.864). The diagnostic value of KANK2 across these tumors is illustrated in Figure 3 A-H. For the remaining cancers, the ROC curves are provided in the Figure S1 for a comprehensive view of KANK2's diagnostic performance.

### Survival analysis of KANK2 in the 33 cancers

To assess the prognostic value of KANK2, we conducted Kaplan-Meier analysis, with results presented in Figure 3 I, J, K. Cox regression analysis of the 33 cancers indicated that KANK2 expression in four cancers was significantly associated with overall survival (OS) (Figure 3 L-O). The results demonstrated that high KANK2 expression correlated with statistically better OS in KIRC, SARC, and UVM, while low KANK2 expression was linked to better OS in LGG. Similar trends were observed for disease-specific survival (DSS), with high KANK2 groups showing better DSS in KIRC and UVM, and low KANK2 groups demonstrating better DSS in LGG (Figure 3 P-R). Additionally, we analyzed progression-free survival (PFS) across the 33 cancers, with significant associations found in ACC and KICH. Specifically, high KANK2 expression was linked to worse PFS in ACC and better PFS in KICH (Figure 3 S-V). Overall, KANK2 appears to play a protective role in KIRC, SARC, UVM, and KICH, while it acts as a risk factor in LGG and ACC.

### KANK2 expression in different immune and molecular subtypes of the 33 cancers

**Figure 4:**
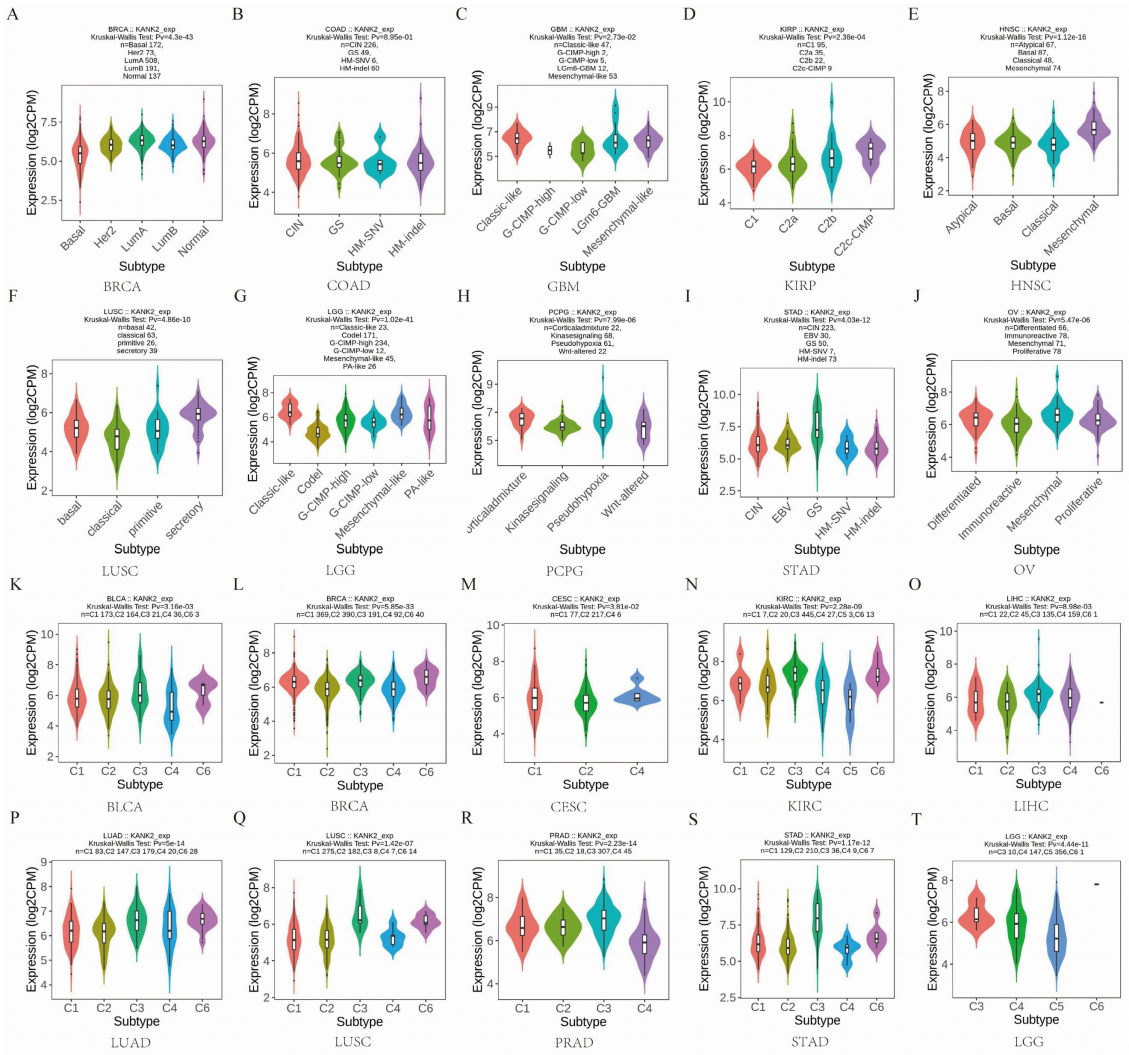
KANK2 expression in different immune and molecular subtypes of the 33 cancers. A-J show molecular subtypes, K-T show immune subtypes.

Given the impact of KANK2 expression on OS in four cancers, we analyzed its expression across immune and molecular subtypes of these and 29 other cancers. Results indicated significant differences in KANK2 expression across immune subtypes in KIRC (6 subtypes) and LGG (3 subtypes) (Figure 4 N, T). For the remaining 29 cancers, notable differential expression of KANK2 was observed in the molecular subtypes of BRCA, COAD, GBM, KIRP, HNSC, LUSC, LGG, PCPG, STAD, and OV (Figure 4 A-J), as well as in immune subtypes of BLCA, BRCA, ESCA, CESC, LIHC, LUAD, LUSC, MESO, OV, PCPG, PRAD, STAD, and UCEC (Figure 4 K-T). In addition, using the TIMER2.0 database, we further analyzed the relationship between KANK2 expression and immune cell infiltration across the 33 cancers. Significant correlations were found with various immune cells, such as CD8+ T cells, CD4+ T cells, macrophages, etc., indicating that KANK2 might be involved in modulating the immune microenvironment of different tumors (Figure S2). These findings provide a more comprehensive understanding of how KANK2 interacts with the tumor immune landscape, particularly in cancers with prominent immune features such as HCC and PAAD.

### Genetic alteration of KANK2

**Figure 5:**
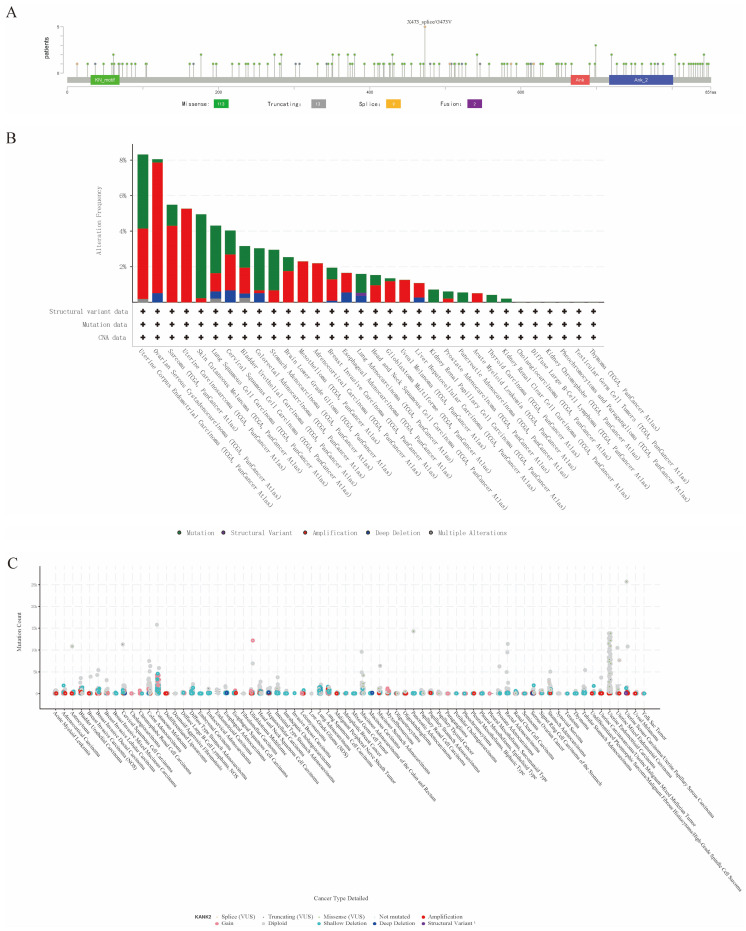
Genetic alteration of KANK2. A shows a lollipop plot of KANK2 gene mutations, and B shows the expression of KANK2 mutation types in different tumors. C shows the mutation counts and types of KANK2 in 70 cancers.

Genetic mutations affecting KANK2 expression were analyzed using the cBioPortal online tool, encompassing all TCGA Pan-Cancer Atlas studies with 13,863 samples. A total of 137 mutation sites were identified within amino acids 0 to 851, including 113 missense mutations, 13 truncating mutations, 9 splice mutations, 2 fusions, with G473V identified as the most frequent mutation site (Figure 5 A). The predominant mutation types were missense and truncating mutations. KANK2 mutations were most commonly observed in UCEC, STAD, melanoma, COAD, READ, and HNSC (Figure 5 B). Among 69 tumor types, shallow deletions, diploid states, and amplifications were common in KANK2 mRNA expression (Figure 5 C).

### Correlation of KANK2 expression with DNA methylation

**Figure 6:**
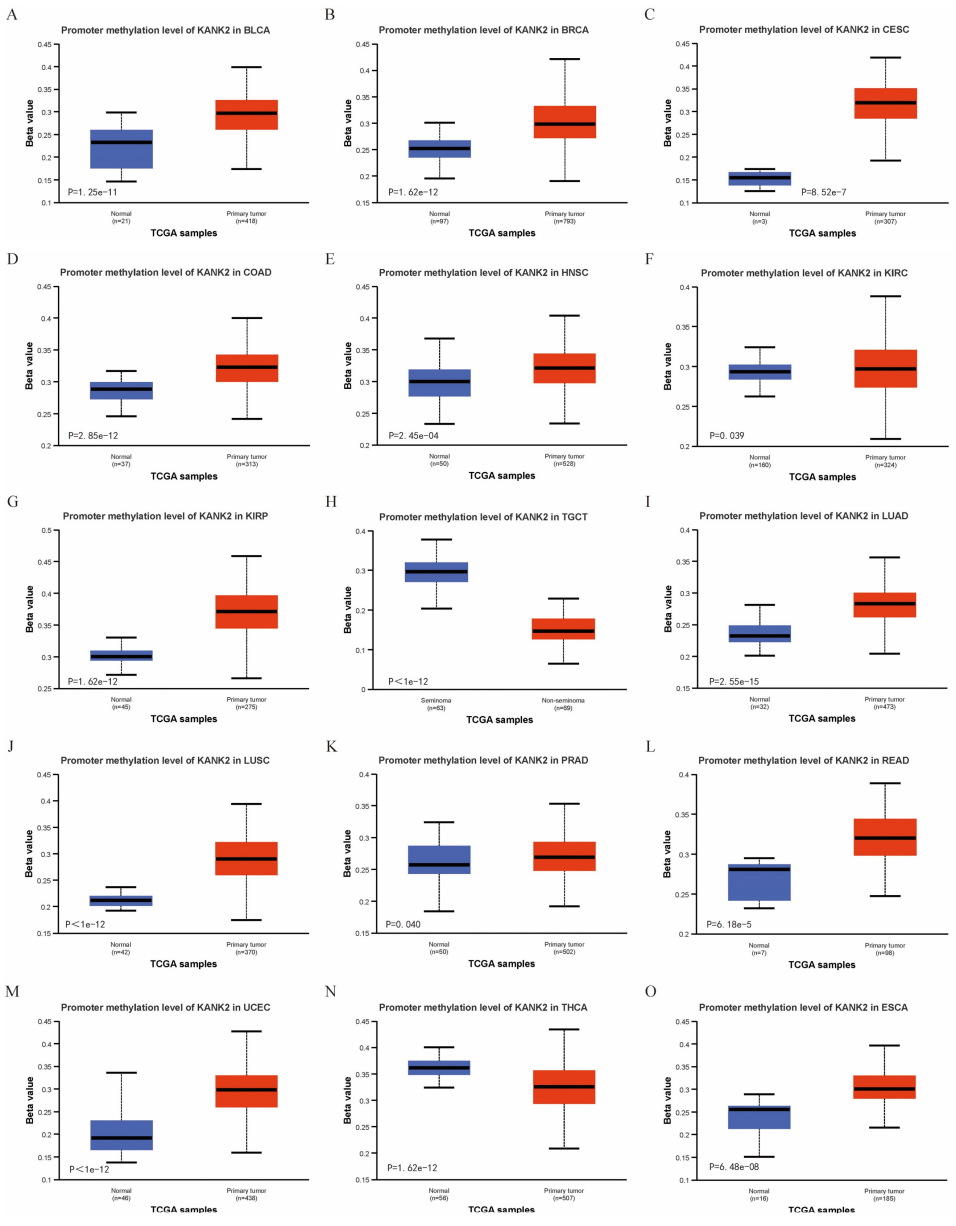
Correlation of KANK2 expression with DNA methylation. A-O show the promoter methylation level of KANK2 was significantly increased in 15 tumor groups compared with the corresponding normal group.

Using the UALCAN online tool, we assessed KANK2 promoter methylation levels across various patient and normal groups for different cancer types. The beta value indicates DNA methylation levels, ranging from 0 (no methylation) to 1 (complete methylation). Specific beta value cutoffs signify hypermethylation (0.7-0.5) or hypomethylation (0.3-0.25). Figure 6 A-O illustrates significantly elevated KANK2 promoter methylation levels in 15 tumor groups compared to their corresponding normal groups.

### Correlations between KANK2 and functional states in different single-cell datasets

**Figure 7:**
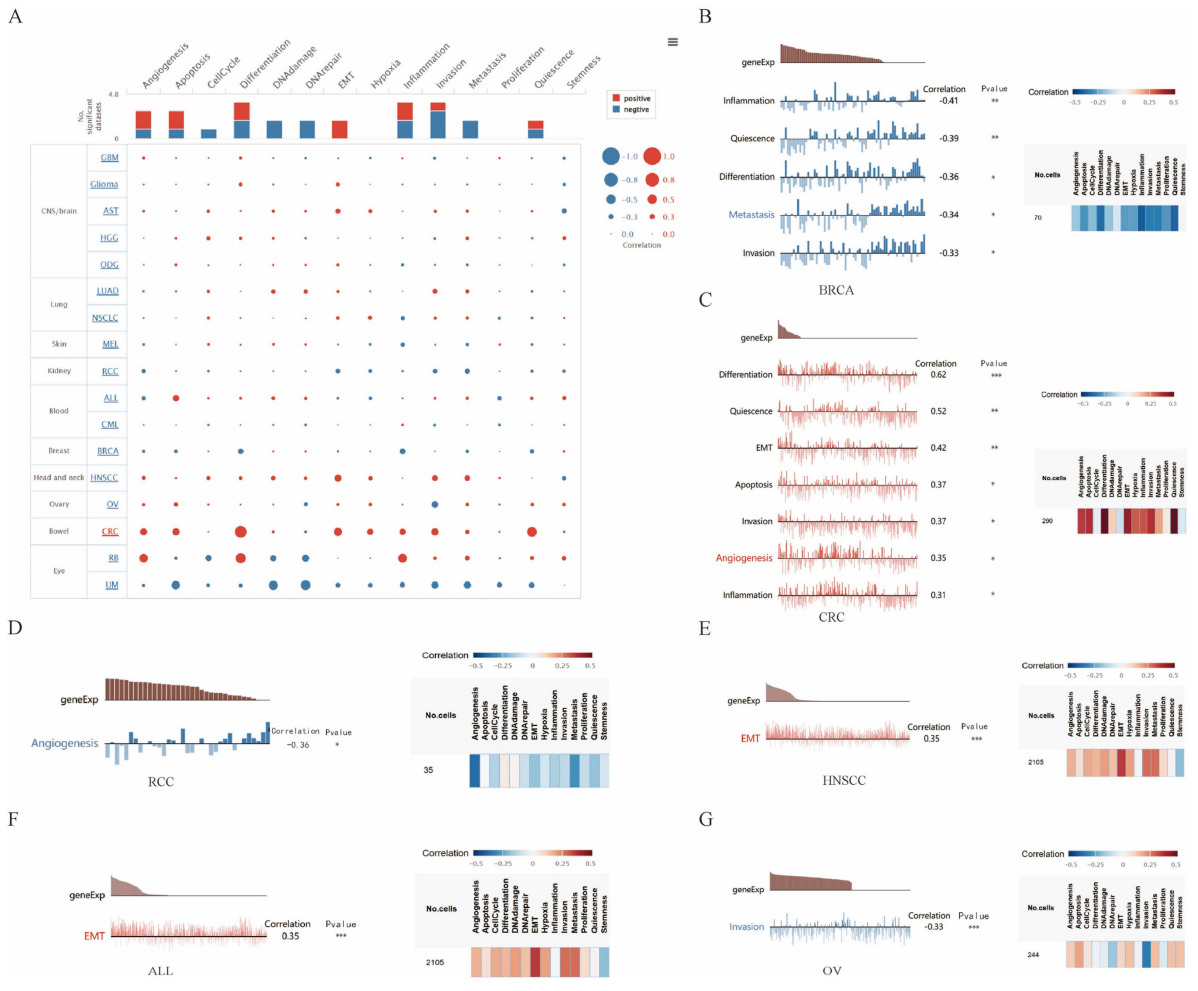
The correlation of KANK2 with functional state in cancers. A shows the interactive bubble chart present correlation of KANK2 with functional state in 17 cancers. B-G demonstrate the relationship between KANK2 and various functional states in BRCA, CRC, RCC, HNSCC, ALL, OV. ***p < 0.001, **p < 0.01, *p < 0.05.

We explored the functional role of KANK2 across various cancer types using the CancerSEA database, which examines KANK2's association with 14 distinct functional states at single-cell resolution. Findings indicated a positive correlation between KANK2 expression and several functional states, including epithelial-mesenchymal transition (EMT), angiogenesis, apoptosis, differentiation, and inflammation. Conversely, negative correlations were observed with DNA damage, DNA repair, metastasis, cell cycle progression, and invasion, with some negative correlations interacting with positive effects (Figure 7 A).

Further assessment revealed that KANK2 positively correlated with differentiation, quiescence, EMT, apoptosis, invasion, angiogenesis, and inflammation in colorectal cancer (CRC). Additionally, a positive association with EMT was noted in head and neck squamous cell carcinoma (HNSCC) and acute lymphoblastic leukemia (ALL). In contrast, KANK2 exhibited negative correlations with inflammation, quiescence, differentiation, metastasis, and invasion in breast cancer (BRCA), as well as with angiogenesis in renal cell carcinoma (RCC) and invasion in ovarian carcinoma (OV) (Figure 7 B-G).

### Gene Set Enrichment Analysis (GSEA)

GSEA results for 15 cancers are shown in Figure S3 A-O, identifying common enrichment pathways including SRP-dependent co-translational protein targeting to membranes, MET activates PTK2 signaling, ribosome-related processes, and ECM receptor interactions. These pathways are associated with biological changes related to proliferation, migration, angiogenesis, and extracellular matrix remodeling, potentially linked to tumor development, tissue repair, and inflammatory responses.

### The PPI, GOKEGG, gene set enrichment and verification

**Figure 8:**
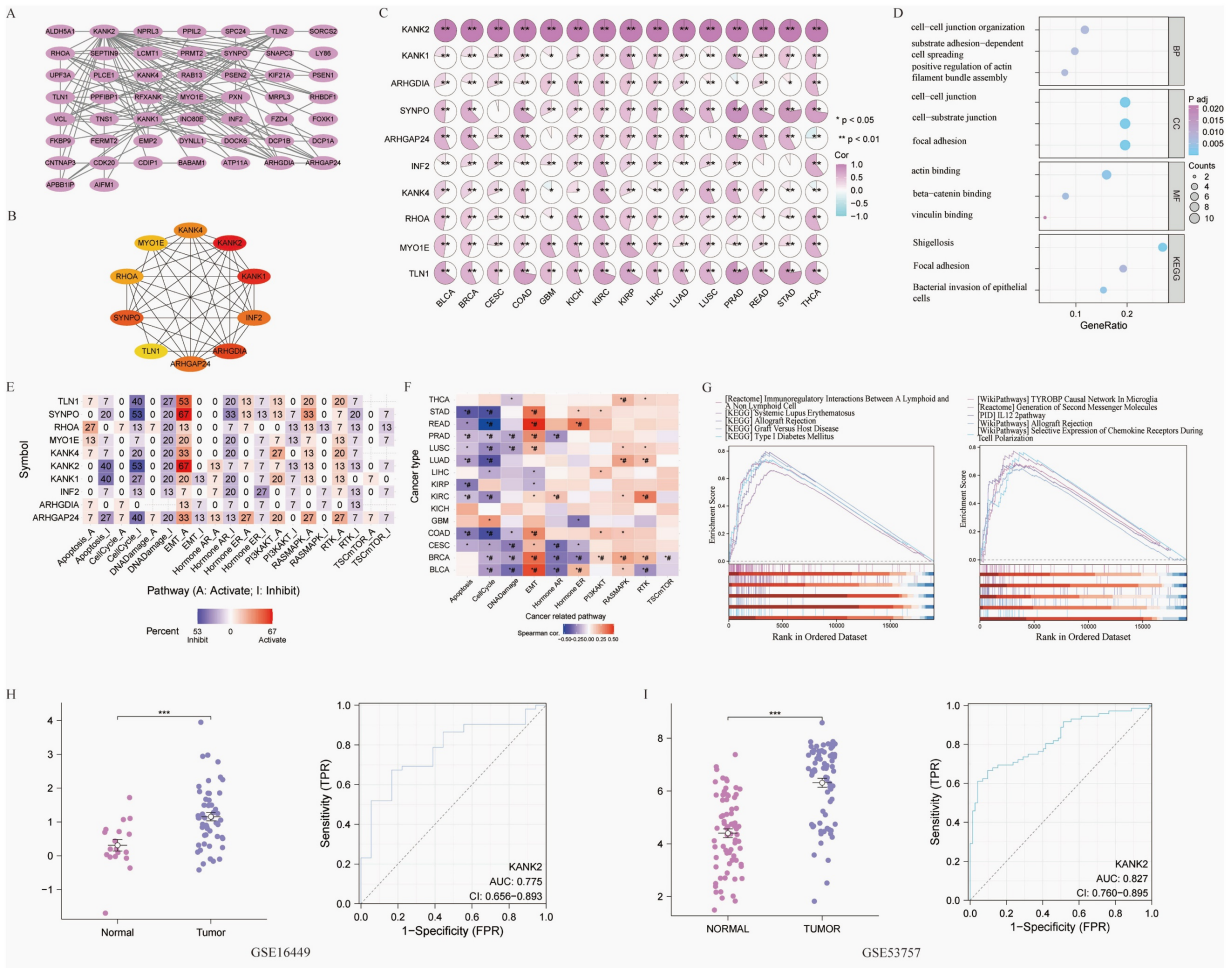
PPI network and Gene set enrichment analysis (GSEA) and validation. A shows the PPI network of HELLS, B shows the top 10 hub genes of PPI network, C shows the association hub gene with KANK2 in 15 cancers present as heatmap. ∗p < 0.05, ∗∗p < 0.01, D shows GOKEGG enrichment. E shows KANK2 with pathway activity or inhibition. F shows heatmap of 15 types of cancer with pathways. G shows GSEA of KIRC RNA sequencing data from TCGA database. H-I show group comparisons and ROC diagnostic curves of 2 datasets (GSE16449 and GSE53757) from the GEO database.

Fifty genes closely linked to KANK2 were obtained from STRING, leading to the construction of a PPI network (Figure 8 A). The top 10 hub genes include KANK2, KANK1, ARHGDIA, SYNPO, ARHGAP24, INF2, KANK4, RHOA, MYO1E, and TLN1 (Figure 8 B). The expression heat map of these hub genes across 15 tumors shows statistically significant relationships (P < 0.01) (Figure 8 C). GO/KEGG enrichment analyses identified key biological processes (BP), molecular functions (MF), and cellular components (CC) associated with these genes, highlighting roles in cell-cell junction organization and integrin interactions (Figure 8 D). KANK2's activation pathways, primarily in cancers, included EMT, hormone AR, RASMAPK, and RTK, while inhibition pathways encompassed the cell cycle, apoptosis, and PI3K/AKT (Figure 8 E).

The correlation between pathway activation and inhibition varied across tumor types; for example, EMT was positively correlated with READ, BLCA, BRCA, PRAD, and KIRC, whereas cell-cycle inhibition was negatively correlated with READ, COAD, STAD, LUAD, and KIRC (Figure 8 F).

Based on ROC and KM curve analyses, we explored the correlation between KANK2 expression and the pathological stage, T stage, and N stage in 33 tumor types, with KIRC showing the strongest association (Figure S4). RNA sequencing data specific to KIRC were retrieved from TCGA for further GSEA, revealing key pathways (Figure 8 G). Two datasets from the GEO database (GSE16449 and GSE53757) were utilized for validation, yielding significant comparisons and ROC diagnostic curves (P < 0.001, AUC > 0.75) (Figure 8 H, I).

### Drug and molecular docking

**Figure 9:**
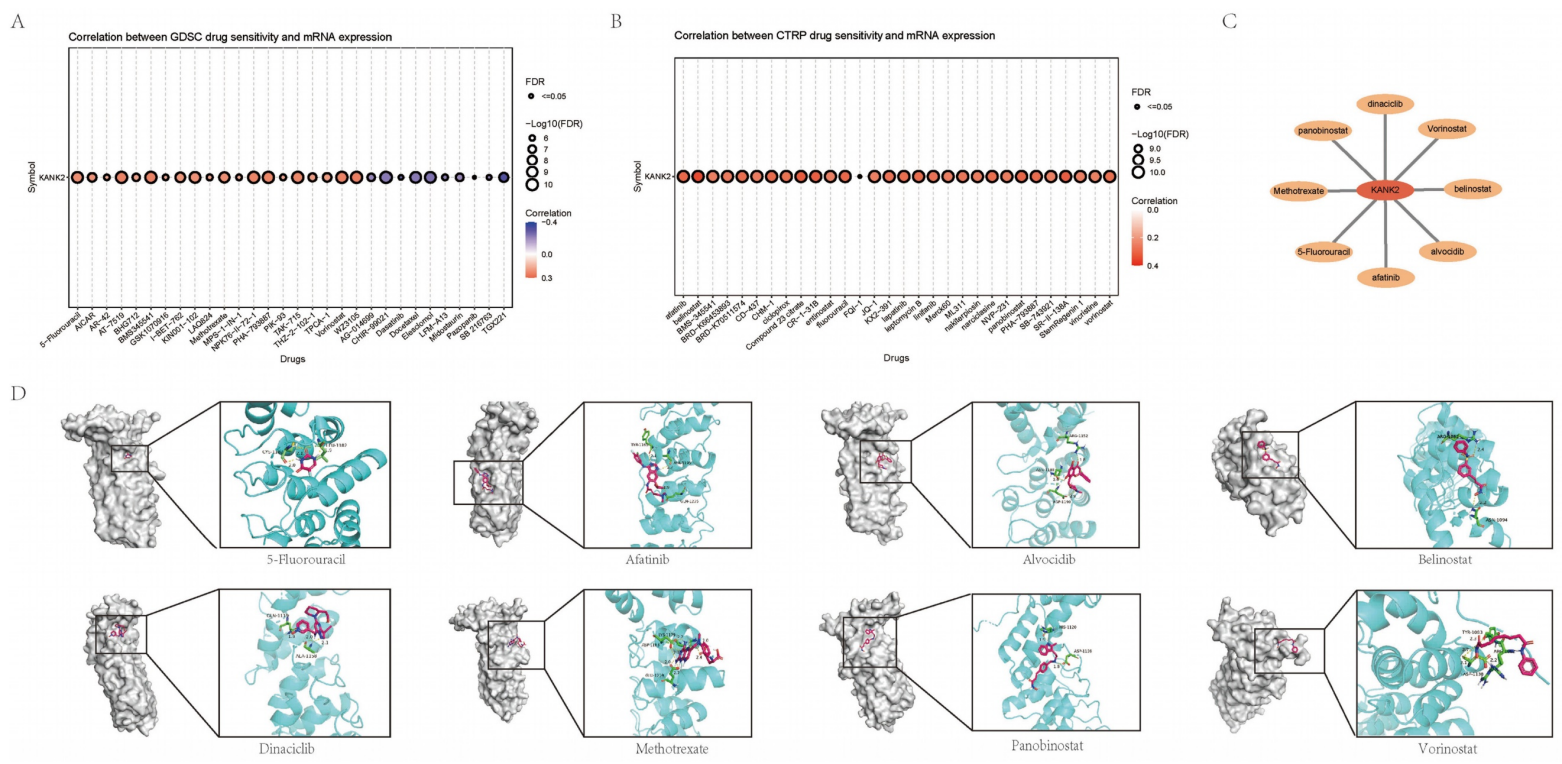
Drug sensitivity and molecular docking. A-B show the most sensitive drugs to KANK2 analyzed from CTRP and GDSC data, respectively. C shows the eight most related drugs to the constructed KANK2. D shows the docking details of the drug molecules.

Using CTRP and GDSC databases via GSEAlite, we identified drugs most related to KANK2 (Figure 9 A-B). The top eight drug molecules included Belinostat, Dinaciclib, Panobinostat, Alvocidib, Afatinib, Vorinostat, 5-Fluorouracil, and Methotrexate, with their relationships to KANK2 illustrated in Cytoscape (Figure 9 C). Molecular docking was performed using Chem3D, Autodock, and Pymol, with visualization of the compound exhibiting the highest binding energy (Figure 9 D).

### Immunohistochemistry, western Blotting, and qPCR

**Figure 10:**
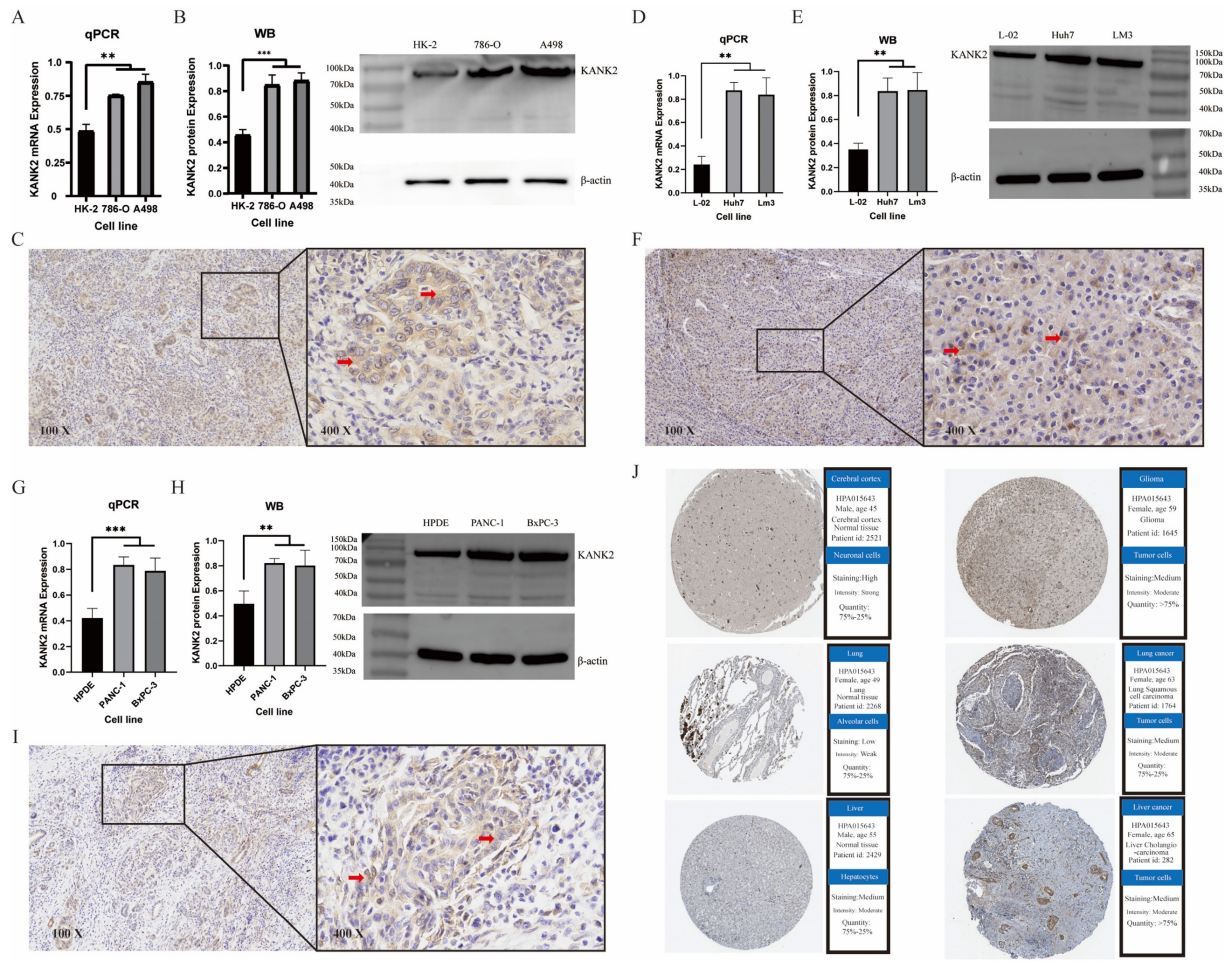
Immunohistochemistry, WB, qPCR experimental validation and validation in the HPA database. A-C show qPCR, WB and IHC staining for KANK2 in KIRC, D-F show qPCR, WB and IHC staining for KANK2 in HCC, and G-I show qPCR, WB and IHC staining for KANK2 in PAAD, and the RED arrow is the positive expression of KANK2 protein. KANK2 protein was mainly expressed in the cytoplasm. J shows the expression of KANK2 in normal and tumor cells from the HPA database. (∗p < 0.05, ∗∗p < 0.01, ∗∗*p < 0.001).

To validate the expression of KANK2 in tumors, we performed immunohistochemistry (IHC), Western blotting (WB) and qPCR analysis in KIRC, HCC and PAAD, respectively. Each group of tumors included one normal cell line and two tumor cell lines. Both WB and qPCR results showed that KANK2 mRNA (Figure 10 A, D, G) and protein levels (Figure 10 B, E, H) were significantly increased in tumor cell lines compared with normal cells (P < 0.05). IHC results showed that KANK2 protein was mainly localized in the cytoplasm (Figure 10 C, F, I). Additionally, IHC data from the Human Protein Atlas (HPA) database corroborated the elevated expression of KANK2 (Figure 10 J).

## Discussion

The KANK protein family, consisting of four members (Kank1-4), is characterized by an N-terminal motif (KN), multiple central coiled-coil domains (CC), and C-terminal ankyrin (Ank) repeats^3^. These proteins are integral to cell adhesion, which is crucial for tumor cell proliferation and epithelial-mesenchymal transition^15^. Specifically, KANK2 plays a significant role in coordinating cell-ECM interactions and microtubule dynamics, facilitating integrin-mediated force transfer and microtubule recruitment^16^-^18^. Aberrant expression of KANK2 in various cancers suggests its involvement in oncogenic pathways^4^, ^19^. highlighting the need for comprehensive pan-cancer analyses to explore its diagnostic and therapeutic potential^20^.

A comprehensive investigation into KANK2 expression across diverse cancer types revealed notable patterns of differential expression, which may have significant diagnostic relevance. The expression of KANK2 at both the mRNA and protein levels was observed in various tissues and organs, showing marked differences between normal and cancerous tissues^21^. Specifically, KANK2 mRNA levels were significantly lower in cancers such as BLCA, BRCA, COAD, KICH, KIRP, LUAD, LUSC, PRAD, READ, and UCEC, whereas higher levels were detected in CHOL, GBM, HNSC, KIRC, LIHC, and PAAD. These results suggest that KANK2 may serve as a valuable biomarker for these cancers. Further support comes from ROC curve analysis, which indicates high diagnostic value for KANK2, with AUC values greater than 0.75 in 13 cancer types, including BLCA, BRCA, and CESC. In PAAD, our analysis shows that KANK2 has an AUC of 0.578, which is lower compared to the widely used biomarker CA19-9, whose AUC is 0.84^22^. This indicates that KANK2 alone may not have sufficient diagnostic power compared to CA19-9. However, it is worth exploring the potential for KANK2 to serve as a supplementary marker, particularly in cases where CA19-9's sensitivity is insufficient, to enhance diagnostic accuracy. In HCC, KANK2 achieved an AUC of 0.794, which is comparable to the AUC of AFP (0.80) as reported in the literature^23^. This suggests that KANK2 could serve as an alternative or complementary biomarker to AFP in HCC diagnosis. Given AFP's well-established use, KANK2 might provide additional biological insights or serve as a complementary biomarker, especially for patients where AFP does not yield definitive results. In addition to PAAD and HCC, KANK2 has also demonstrated diagnostic potential across multiple other cancer types. Our study revealed that KANK2 is widely expressed in various cancers and exhibits significant diagnostic value in some of them, as indicated by ROC curve analysis. For instance, in cancers such as lung adenocarcinoma (LUAD) and colorectal cancer (CRC), KANK2 showed moderate AUC values, suggesting its utility in broader cancer diagnostics. This highlights the versatility of KANK2 as a biomarker, which could be valuable either independently or as part of a panel of biomarkers, improving diagnostic specificity and sensitivity across different types of cancers.

Kaplan-Meier and Cox regression analyses demonstrated that elevated KANK2 expression correlates with improved overall survival (OS) and disease-specific survival (DSS) in cancers such as KIRC, SARC, and UVM. Conversely, lower KANK2 expression was associated with better survival outcomes in LGG. These results suggest that KANK2 has a complex role in cancer prognosis, acting as a protective factor in some cases, while serving as a risk factor in others. Some researchers have suggested that this may be due to variations in the tumor microenvironment^24^, ^25^, different tumor development stages^26^, or tumor cell heterogeneity^27^. Understanding the mechanisms underlying KANK2's dual role is essential for developing targeted therapies that modulate its expression to improve patient outcomes.

Our exploration of KANK2 expression across different immune and molecular subtypes of cancer revealed significant variations, suggesting potential implications for personalized cancer treatments^28^, ^29^. KANK2 expression showed significant differences in immune subtypes of KIRC and LGG, as well as in BLCA, BRCA, and LIHC. These findings imply that KANK2 expression is influenced by the tumor microenvironment and immune landscape^30^, ^31^, which may impact immunotherapy responses. Additionally, the variable expression of KANK2 in molecular subtypes like Basal^32^, Her2^33^, ^34^, and lumA^35^, further emphasizes its potential utility as a biomarker for patient stratification and treatment optimization. In our expanded analysis of KANK2, we evaluated its role in immune infiltration in melanoma and HCC. Using the TIMER and TISIDB databases, we found that KANK2 expression is significantly correlated with various immune cells, including CD8+ T cells and macrophages in melanoma. These findings suggest that KANK2 may play a role in modulating the tumor immune microenvironment, potentially affecting the efficacy of immunotherapies in these cancers. Furthermore, we explored the correlation between KANK2 and immune checkpoints such as PD-1, PD-L1, and CTLA-4. In HCC, KANK2 expression was found to be positively correlated with these immune checkpoint markers, indicating that KANK2 may contribute to the formation of an immunosuppressive environment, which could influence the effectiveness of immunotherapy. These results provide new insights into the potential clinical applications of KANK2 in hepatobiliary cancers, particularly as immunotherapy becomes increasingly adopted. Overall, these findings indicate that KANK2 may impact tumor biology across various cancers by modulating the immune microenvironment and holds potential as a target for immunotherapy. KANK2 expression is significantly correlated with the infiltration levels of immune cells such as CD8+ T cells and macrophages in melanoma and NSCLC, which are crucial for the response to immunotherapy^36^, ^37^. Therefore, combining KANK2 inhibition with immune checkpoint inhibitors (such as PD-1/PD-L1 blockers) may produce synergistic effects, enhancing the efficacy of immunotherapy in these cancers. Similarly, combining KANK2 inhibitors with conventional chemotherapy agents could improve treatment outcomes in hepatobiliary cancers, such as HCC.

The genetic alteration analysis of KANK2 revealed a high frequency of mutations, especially missense and truncating mutations, in cancers such as UCEC, STAD, and KIRC. These mutations may disrupt KANK2's normal function, contributing to cancer development and progression. M.P. Ramirez *et al.* suggested that missense mutations might lead to decreased focal adhesion tension, which could explain KANK2's role as a proto-oncogene^38^. Additionally, our DNA methylation analysis showed significantly higher KANK2 promoter methylation levels in tumors compared to normal tissues. Promoter hypermethylation may lead to gene silencing, affecting the tumor microenvironment and drug resistance to immunotherapy^39^, ^40^, which aligns with this study's results. The relationship between KANK2 mutations and methylation status with cancer progression underscores the importance of these alterations in regulating KANK2 expression. Future studies should aim to clarify the functional consequences of these alterations and explore their therapeutic potential.

Functional state analysis using CancerSEA revealed that KANK2 is significantly correlated with several cancer-related processes, such as EMT, differentiation, angiogenesis, metastasis, invasion, apoptosis, and inflammation. These results suggest that KANK2 is a critical player in the regulation of processes that drive tumor progression and metastasis, consistent with findings by Marija Lončarić *et al.*^5^, ^19^. The GOKEGG analysis and gene set enrichment analysis (GSEA) further supported these findings. Through the 10 hubgenes screened by string database, the relationship between 10 hubgenes and 15 tumors was made, and the GOKEGG analysis showed that KANK2 played a role in cell migration, cytoskeleton reorganization, and cell signal transduction, which was consistent with the results of previous studies^5^, ^6^, ^19^, ^41^. It may affect the migration and invasion ability of tumor cells by regulating the cytoskeleton and cell adhesion molecules, affect the metastasis and invasion of tumor cells, regulate the signaling pathways that affect cell cycle and proliferation, thus affecting the proliferation rate of tumor cells, and also affect the interaction between cells in the tumor microenvironment. Including the interaction of tumor cells with immune cells and stromal cells, in turn, it affects tumor development and immune escape^42^-^45^. The identification of these pathways provides valuable insights into the biological functions of KANK2 and highlights potential targets for therapeutic intervention.

Through the analysis results of 33 tumors in TCGA database, we can see that KANK2 is not only differentially expressed in KIRC (P < 0.01), but also its prognostic KM curve and diagnostic ROC curve are statistically significant. Two data sets were found and verified in GEO database. It was found that the group comparison and diagnostic ROC were still statistically significant, indicating that KANK2 can be used as a biomarker for the diagnosis and prognosis of KIRC. To verify KANK2 expression at the protein and mRNA levels, we conducted qPCR, Western blot (WB), and immunohistochemistry (IHC) experiments in KIRC, HCC, and PAAD cell lines. The results were consistent across these three cancers, demonstrating that KANK2 expression is aligned with previous findings, thereby confirming its potential significance as a biomarker. In order to further investigate the role of KANK2 in drug therapy, we identified compounds highly correlated with KANK2 expression through analysis of the CTRP and GDSC databases using the online platform GSCALite. These compounds included 5-fluorouracil (5-FU), Belinostat, Dinaciclib, and Panobinostat. Molecular docking revealed strong binding interactions between KANK2 and these compounds, involving different mechanisms such as cell cycle control, DNA synthesis, and signaling pathways that inhibit tumor cell growth and proliferation^46^-^48^. These findings further confirm that KANK2 can be targeted for tumor drug therapy, offering hope for future treatment strategies in cancers like KIRC, HCC, and PAAD.

## Conclusion

Our investigation into KANK2 expression across various cancer types, along with its involvement in signaling pathways, mutation sites, promoter DNA methylation, immune infiltration, GOKEGG pathway analyses, and drug associations, has illuminated its critical role in cancer biology. The diagnostic and prognostic significance of KANK2, as well as its potential as a therapeutic target, underscores its importance in the context of oncology.

Despite these significant findings, our study has several limitations that need to be acknowledged. First, our reliance on retrospective datasets, primarily from TCGA and GTEx, may introduce selection bias, as these datasets represent a specific subset of the population with distinct clinical and demographic characteristics. This selection bias limits the generalizability of our findings, and future prospective studies are needed to validate the conclusions in more diverse patient cohorts. Second, the experimental validations for KANK2 were conducted primarily in specific cancer types, including KIRC, PAAD, and HCC. While these experiments provided valuable insights, validations across a broader range of cancer types are necessary to further confirm KANK2's role as a pan-cancer biomarker. Expanding the experiments to other cancers, such as lung and colorectal cancers, would help strengthen the general applicability of our results. Finally, although our study focused on bioinformatics analyses combined with experimental validation, further clinical validation through larger-scale patient studies and clinical trials is required to determine KANK2's real-world applicability in diagnosis, prognosis, and therapeutic targeting.

Addressing these limitations in future research will provide a more comprehensive understanding of KANK2's biological functions and its potential clinical applications across different cancers. Prospective studies, experimental validation in diverse cancer types, and mechanistic investigations are essential to deepen our understanding of KANK2's functional roles and therapeutic potential, ultimately enhancing its translational impact as a biomarker and therapeutic target in oncology.

## Supplementary Material

Supplementary figures.
