# Supplementary material for: Pan-Cancer Analysis of KANK2: Clinical and Molecular Insights into Tumor Progression and Therapeutic Implications
Source: J Cancer. 2025 Jan 6;16(4):1149–66. doi: 10.7150/jca.105098 (PMC11786046; doi:10.7150/jca.105098)
Supplement: Supplementary file 1 — Supplementary figures. [file jcav16p1149s1.pdf]

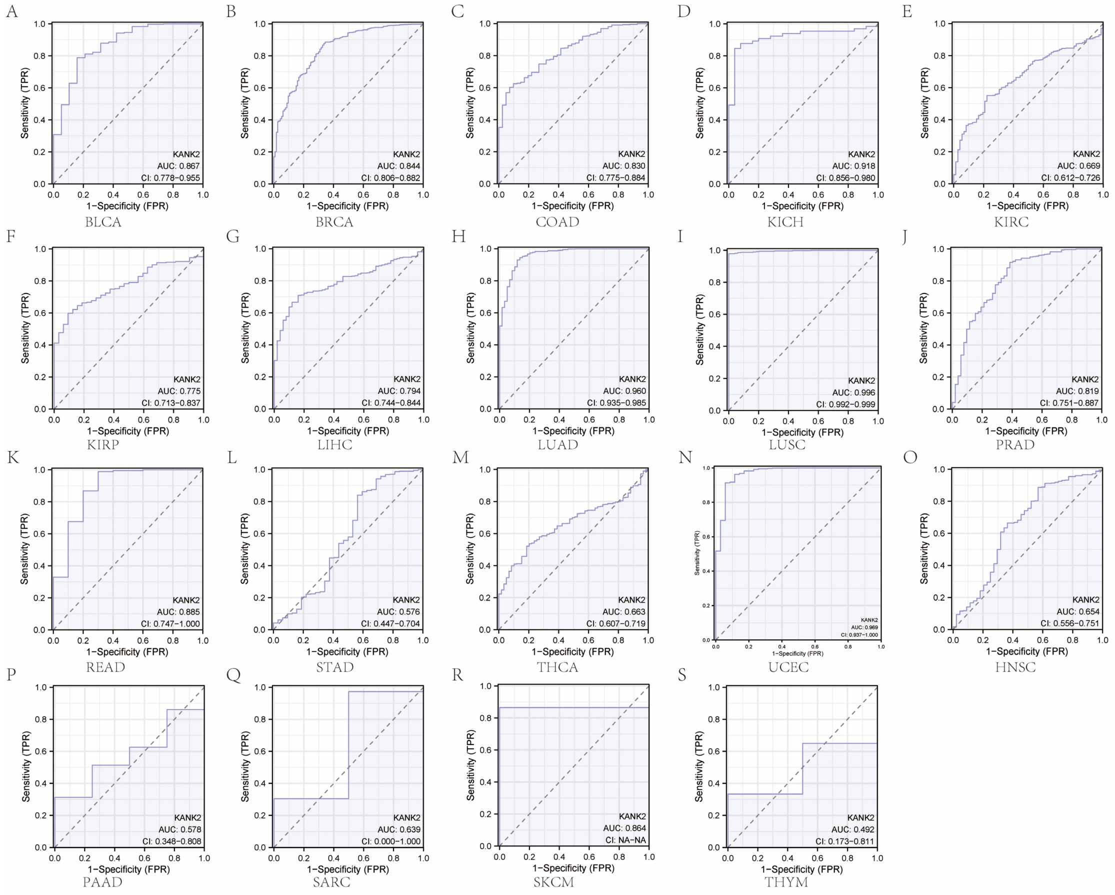

Figure S1 ROC curve expression in 19 tumors. ROC curves are presented for 19 cancer types where sufficient data remained after excluding missing values, while other cancers lacked sufficient outcome diversity for ROC analysis.

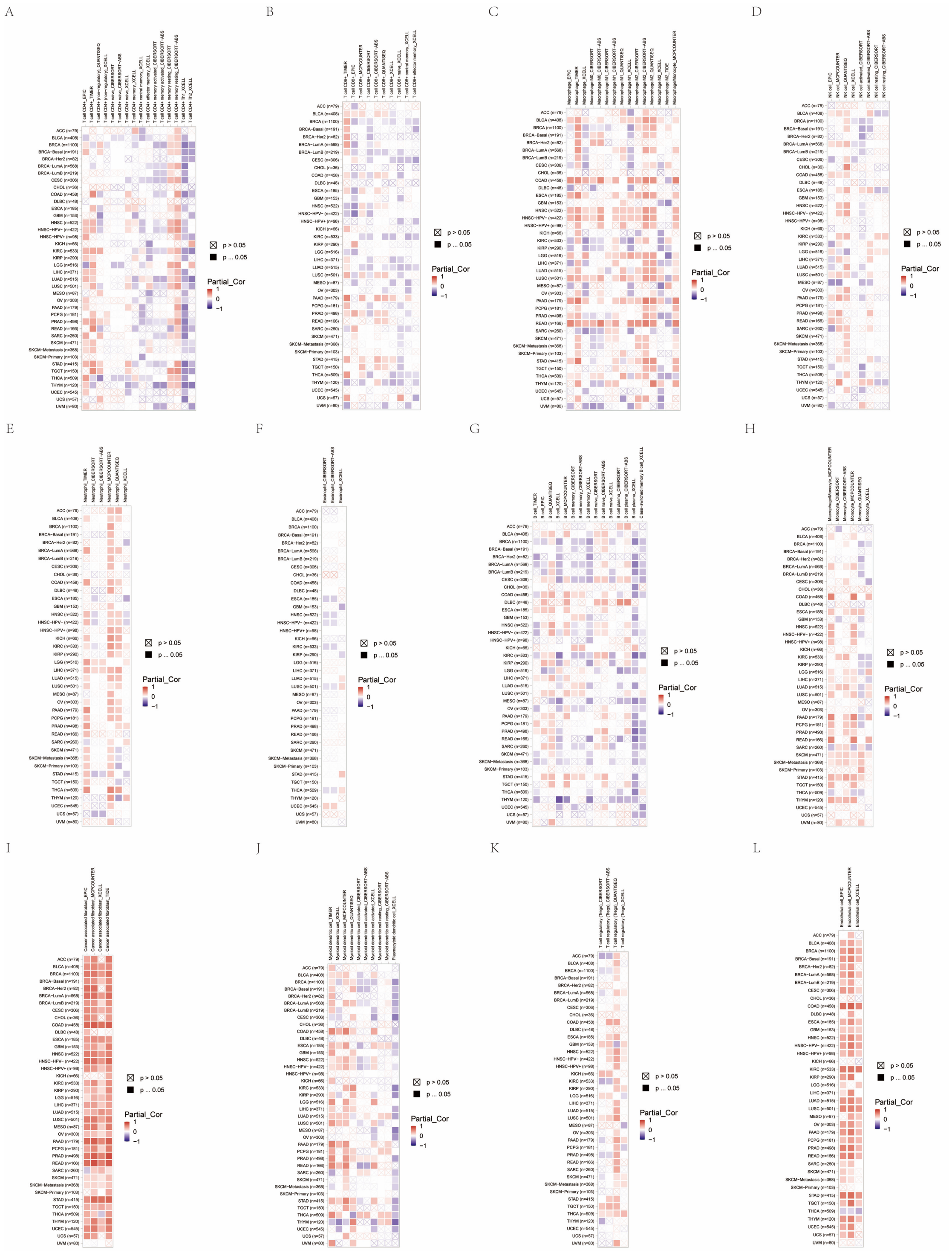

Figure S2 Correlation Between KANK2 Expression and Immune Cell Infiltration in 33 Cancer Types. Correlation analysis between KANK2 expression and various immune cells (e.g., CD4+ T cells, CD8+ T cells, macrophages, NK cells, neutrophils, Eosinophil, B cells, monocytes, cancer-associated fibroblasts, myeloid dendritic cell, Tregs, and endothelial cells) in 33 cancers, based on TIMER2.0 database results.

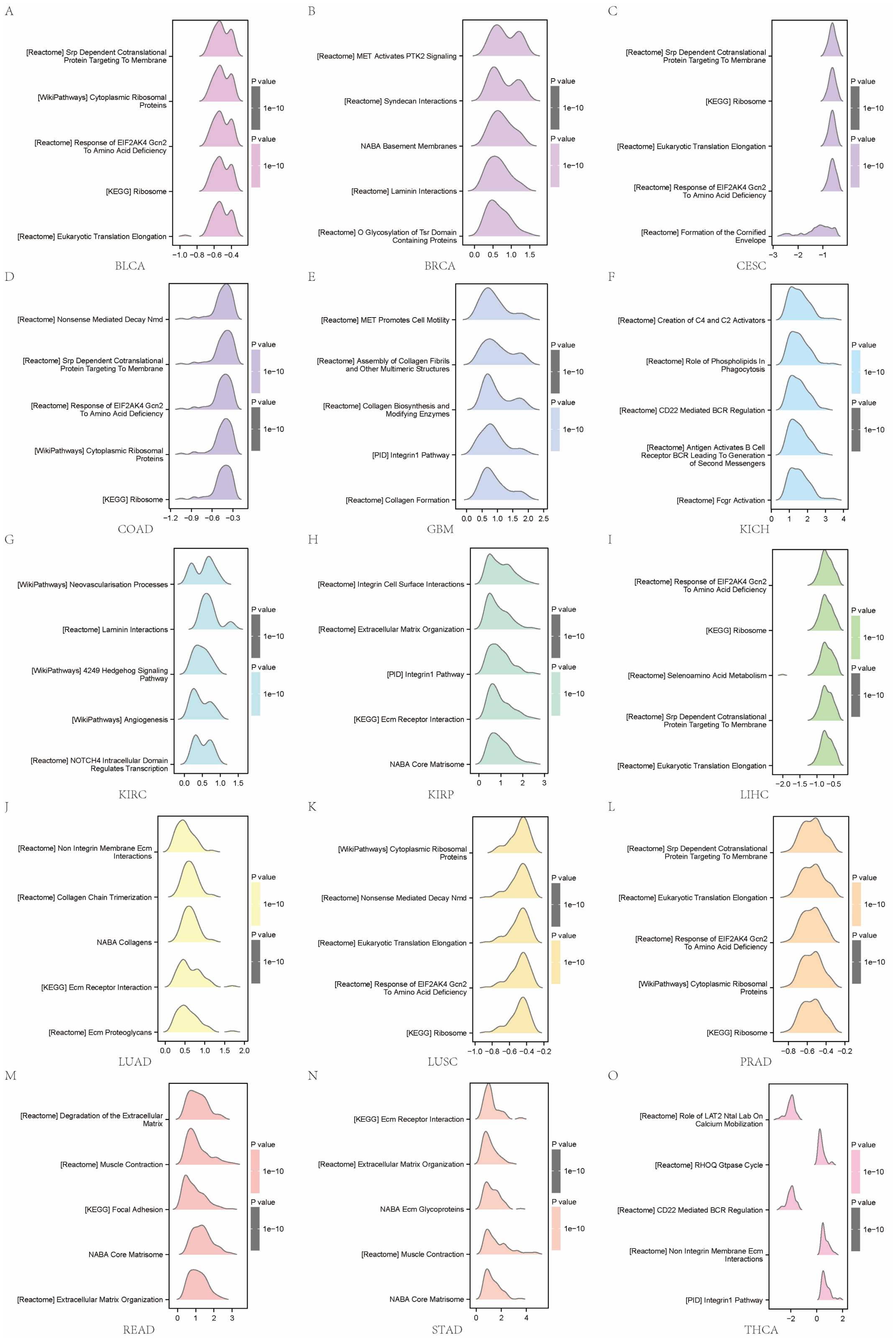
A
[Reactome] Srp Dependent Cotranslational Protein Targeting To Membrane
[WikiPathways] Cytoplasmic Ribosomal Proteins
[Reactome] Response of EIF2AK4 Gcn2 To Amino Acid Deficiency
[KEGG] Ribosome
[Reactome] Eukaryotic Translation Elongation
P value
1e−10
BLCA
B
[Reactome] MET Activates PTK2 Signaling
[Reactome] Syndecan Interactions
NABA Basement Membranes
[Reactome] Laminin Interactions
[Reactome] O Glycosylation of Tsr Domain Containing Proteins
P value
1e−10
BRCA
C
[Reactome] Srp Dependent Cotranslational Protein Targeting To Membrane
[KEGG] Ribosome
[Reactome] Eukaryotic Translation Elongation
[Reactome] Response of EIF2AK4 Gcn2 To Amino Acid Deficiency
[Reactome] Formation of the Cornified Envelope
P value
1e−10
CESC
D
[Reactome] Nonsense Mediated Decay Nmd
[Reactome] Srp Dependent Cotranslational Protein Targeting To Membrane
[Reactome] Response of EIF2AK4 Gcn2 To Amino Acid Deficiency
[WikiPathways] Cytoplasmic Ribosomal Proteins
[KEGG] Ribosome
P value
1e−10
COAD
E
[Reactome] MET Promotes Cell Motility
[Reactome] Assembly of Collagen Fibrils and Other Multimeric Structures
[Reactome] Collagen Biosynthesis and Modifying Enzymes
[PID] Integrin1 Pathway
[Reactome] Collagen Formation
P value
1e−10
GBM
F
[Reactome] Creation of C4 and C2 Activators
[Reactome] Role of Phospholipids In Phagocytosis
[Reactome] CD22 Mediated BCR Regulation
[Reactome] Antigen Activates B Cell Receptor BCR Leading To Generation of Second Messengers
[Reactome] Fcgr Activation
P value
1e−10
KICH
G
[WikiPathways] Neovascularisation Processes
[Reactome] Laminin Interactions
[WikiPathways] 4249 Hedgehog Signaling Pathway
[WikiPathways] Angiogenesis
[Reactome] NOTCH4 Intracellular Domain Regulates Transcription
P value
1e−10
KIRC
H
[Reactome] Integrin Cell Surface Interactions
[Reactome] Extracellular Matrix Organization
[PID] Integrin1 Pathway
[KEGG] Ecm Receptor Interaction
NABA Core Matrisome
P value
1e−10
KIRP
I
[Reactome] Response of EIF2AK4 Gcn2 To Amino Acid Deficiency
[KEGG] Ribosome
[Reactome] Selenoamino Acid Metabolism
[Reactome] Srp Dependent Cotranslational Protein Targeting To Membrane
[Reactome] Eukaryotic Translation Elongation
P value
1e−10
LIHC
J
[Reactome] Non Integrin Membrane Ecm Interactions
[Reactome] Collagen Chain Trimerization
NABA Collagens
[KEGG] Ecm Receptor Interaction
[Reactome] Ecm Proteoglycans
P value
1e−10
LUAD
K
[WikiPathways] Cytoplasmic Ribosomal Proteins
[Reactome] Nonsense Mediated Decay Nmd
[Reactome] Eukaryotic Translation Elongation
[Reactome] Response of EIF2AK4 Gcn2 To Amino Acid Deficiency
[KEGG] Ribosome
P value
1e−10
LUSC
L
[Reactome] Srp Dependent Cotranslational Protein Targeting To Membrane
[Reactome] Eukaryotic Translation Elongation
[Reactome] Response of EIF2AK4 Gcn2 To Amino Acid Deficiency
[WikiPathways] Cytoplasmic Ribosomal Proteins
[KEGG] Ribosome
P value
1e−10
PRAD
M
[Reactome] Degradation of the Extracellular Matrix
[Reactome] Muscle Contraction
[KEGG] Focal Adhesion
NABA Core Matrisome
[Reactome] Extracellular Matrix Organization
P value
1e−10
READ
N
[KEGG] Ecm Receptor Interaction
[Reactome] Extracellular Matrix Organization
NABA Ecm Glycoproteins
[Reactome] Muscle Contraction
NABA Core Matrisome
P value
1e−10
STAD
O
[Reactome] Role of LAT2 Ntal Lab On Calcium Mobilization
[Reactome] RHOQ Gtpase Cycle
[Reactome] CD22 Mediated BCR Regulation
[Reactome] Non Integrin Membrane Ecm Interactions
[PID] Integrin1 Pathway
P value
1e−10
THCA

Figure S3. GSEA functional enrichment analysis of KANK2 expression in 15 cancers. A-O shows the top 5 GSEA functional enrichment pathways of KANK2 in BLCA, BRCA, CESC, COAD, GBM, KICH, KIRC, KIRP, LIHC, LUAD, LUSC, PRAD, READ, STAD, THCA. The Y-axis represents one gene set and the X-axis is the distribution of logFC corresponding to the core molecules in each gene set.

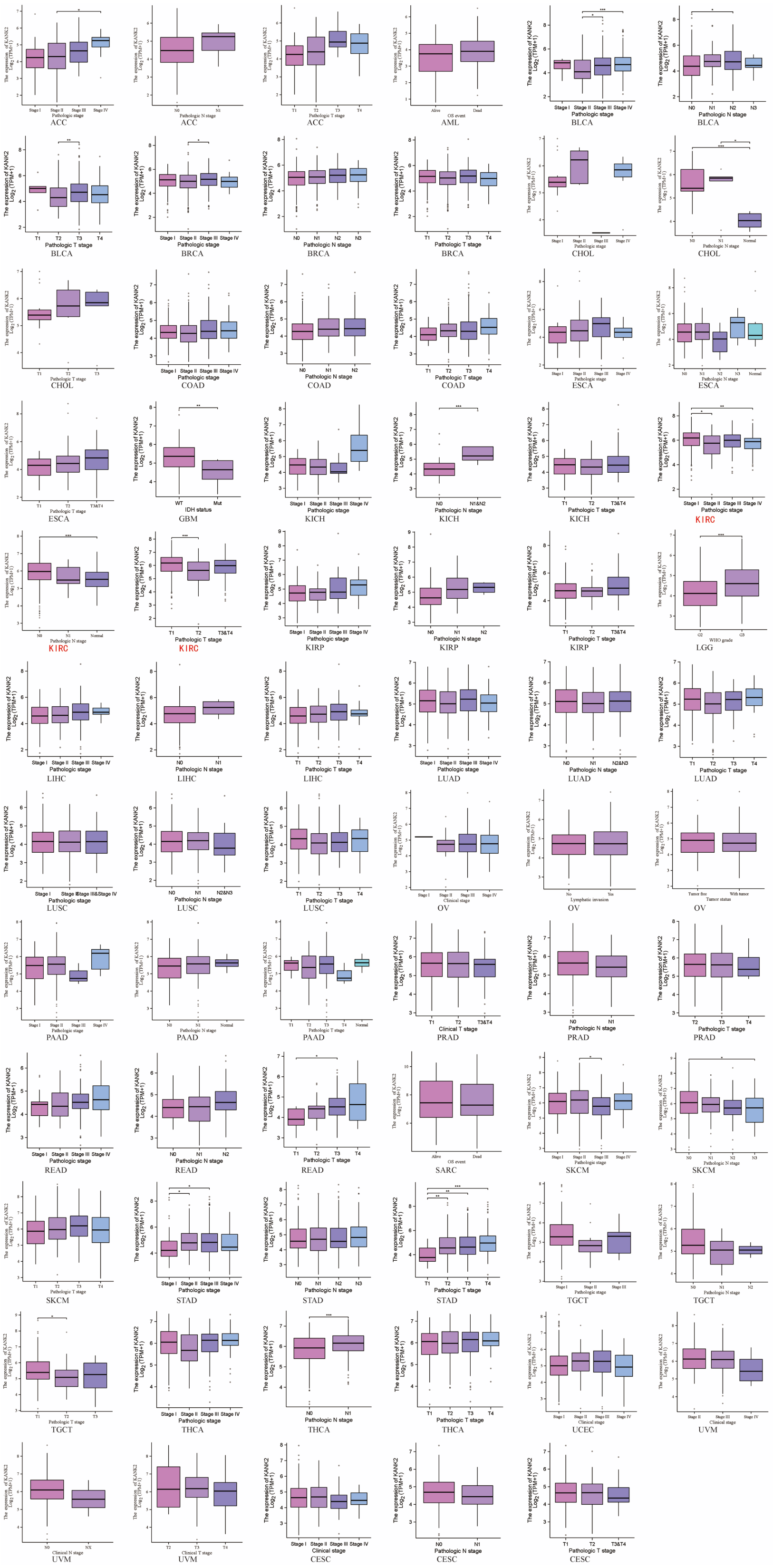

Figure S4. The correlation between KANK2 expression and the pathological stage, T stage, and N stage of 33 tumor types. Kidney Renal Clear Cell Carcinoma (KIRC) demonstrated the strongest association with diagnostic ROC, prognostic KM curves, and staging parameters.
